# The mediating effect of force and velocity changes on power output enhancement in bench press throw after submaximal isometric conditioning activity in trained males

**DOI:** 10.5114/biolsport.2025.142639

**Published:** 2024-08-30

**Authors:** Dawid Koźlenia, Michał Krzysztofik

**Affiliations:** 1 Unit of Biostructure, Faculty of Physical Education and Sport, Wroclaw University of Health and Sport Sciences; 2 Institute of Sport Sciences, Jerzy Kukuczka Academy of Physical Education in Katowice, Poland

**Keywords:** Physical performance, Upper extremities, Muscles, Mediation analysis, Kinematics

## Abstract

This study examined the mediating effect of force and velocity changes on power output enhancement after isometric submaximal conditioning activity (CA) in trained males. Males aged 19–25 years, with at least three years of continuous resistance training, were randomly assigned to experimental (EG; n = 14) and control (CG, n = 12) groups. The CA consisted of four three-second sets of isometric bench presses, using a load of 70% one-repetition maximum (1RM) with a 60-second rest interval. Bench press throw (BPT) was performed at 30% 1RM at baseline (3 minutes before CA) and at 1, 4, 7, and 10 minutes after the CA. Set mean velocity (sMV), set mean force (sMF), and set mean power output (sMP) were analyzed. A repeated measures ANOVA revealed significant improvements in sMV (F = 6.10; p < 0.01) and sMP (F = 5.03; p < 0.01) with no significant differences in force values (F= 0.47; p = 0.55). The enhancements in sMV and sMP were observed at 4 minutes after the CA compared to baseline (p < 0.01) and were sustained during the subsequent minutes (p < 0.05). Mediation analysis confirmed the observed improvements in power output were mediated by velocity enhancement (β = 0.37; p < 0.01) with an insignificant impact on force (β = -0.03; p = 0.61). Using a moderate submaximal load in hold isometric muscle action, the examined CA protocol effectively enhanced upper body power output through increased barbell velocity while showing no changes in force production.

## INTRODUCTION

Enhancement in power output can be achieved acutely through the phenomenon called post-activation performance enhancement (PAPE), which is a short-term improvement in performance following a conditioning activity (CA) [1]. Evidence suggests that the mechanisms behind PAPE involve a combination of myosin light chain phosphorylation, an increase in muscle temperature, fluctuations in intramuscular water content, and improvements in neural drive [2, 3]. Rassier and Mcintosh [4] postulated that the occurrence of PAPE is dependent on heightened excitation and reduced fatigue. Since power is a product of force and velocity, analyzing these two parameters would provide more insight into the PAPE effect on power performance [5]. However, to the best of the authors’ knowledge, no studies have provided such an analysis that considers power output improvement through changes in force and velocity and how they contribute to the observed performance enhancement.

Indeed, specific training interventions targeted at force or velocity improvement may enhance power output [6]. Therefore, the question arises of whether an acute intervention may enhance power output specifically through force or velocity. Moreover, a study by Seitz & Haff [7] showed that various types of CA provide different effects, which suggests some possible specificity for PAPE protocols in power improvement. Tillin and Bishop [8] indicated that velocity increases at a given force level (or the opposite) in the PAPE effect. Increased force production due to CA may result from greater motor unit recruitment and rate coding [9]. Velocity improvement is due to faster contraction speeds, also influenced by muscle-tendon stiffness and elastic energy utilization [10]. Enhanced activation of muscle fibers can increase velocity while activating more fibers, and the formation of cross-bridges in the sarcomere elevates force production [11]. Studies indicate that the PAPE effect varies with the nature of the CA, implying it is not uniform and can be modulated by activity type [1, 7]. These reports suggest that changes in velocity and force may independently contribute to improvements in power output.

Most studies are focused on the impact of CAs on subsequent tasks [7, 12], whereas observed improvements in velocity and force production after isometric activity were recorded [13, 14]. This justifies the application of multivariate analysis to examine the impact of CAs on power output in the presence of other variables. This capability can be addressed via mediation analysis [15], which allows for a more profound comprehension of the underlying mechanisms that influence established associations. By exploring the role of a mediator (force and velocity), mediation analysis would show how CAs may impact power output [16].

Recognizing the importance of upper body performance in sports, isometric CAs for the upper body remain largely unexplored [17, 18]. Long-term isometric interventions can enhance muscle strength and tendon properties, reduce injury risk, and improve performance [19]. However, there is limited knowledge about the acute effects of submaximal isometric muscle actions on upper body power output, despite positive outcomes for the lower body were observed [20, 21]. Submaximal isometric muscle actions demand higher nervous system engagement due to the need to match forces with inertia to maintain isometric conditions, while their relatively low energy expenditure may delay fatigue onset [22, 23]. The increased nervous system activity (higher excitation) with low-energy-cost activities results in the PAPE effect [4].

This study examined the mediating effect of force and velocity changes on power output enhancement during the bench press throw (BPT) after isometric submaximal CA in trained males. Specifically, we asked if and how changes in mean power output are specifically affected by changes in mean force and velocity after introducing the CA. We hypothesized that the CA protocol would enhance power output during the BPT through changes in both force and velocity, which would mediate the improvement in power output, but the role of both kinematic parameters would vary. Given the limited available data on PAPE protocols for upper body power performance using isometric CAs, this study is anticipated to contribute novel insights and offer practical strategies for practitioners seeking to leverage the PAPE effect for acute improvements in upper body performance by demonstrating how changes in force and velocity contribute to power output changes after CA.

## MATERIALS AND METHODS

### Ethics

This study adhered to the principles of the Declaration of Helsinki and received approval from the Ethics Committee of the Wroclaw University of Health and Sport Sciences. Participants volunteered after being informed about the study procedures, potential risks, and the option to withdraw at any point. All participants provided written consent.

### Experimental Approach

This study states a randomized controlled trial. Participants supplied details about their training background, bench press (BP) one-repetition maximum (1RM), and injury history. During the initial meeting, participants underwent a standard warm-up, which included a fiveminute treadmill run at 6 km/h, dynamic joint mobilization, three sets of 12–15 repetitions of bodyweight push-ups, and two sets of 15–20 BP repetitions using an empty bar. Subsequently, the participants’ BP 1RM was determined using the force-velocity method, and body morphology measurements were taken. Following this, participants were introduced to the BPT and the PAPE protocol. After a familiarization session, participants were randomly assigned to the experimental group (EG) or control group (CG). During the experimental session, participants in both groups followed the same standard warm-up filled up with 1–2 sets of 3–5 repetitions of BPT at the maximum intended velocity with an empty bar, and then in the following two sets, performed BPT with their 30% 1RM of BP with 90 seconds of rest. Subsequently, a baseline set of three repetitions of BPT was performed at 30% 1RM 3 minutes before the CA. Following this, the EG executed the PAPE protocol while the CG engaged in a five-minute treadmill run at 6 km/h to maintain body temperature and preparedness for the BPT. The CA for the EG consisted of four three-second sets with a 60-second rest interval of isometric holds in BP using 70% of 1RM, with the full bent arms with the barbell actively held in contact with their chest. The handgrip was set at 150% of the biacromial distance. The researchers carefully controlled that no one put the bar on their chest and that the participants maintained a constant maximum tension. Post-activation BPT measurements were recorded 1 minute after CA and then after 4 minutes, 7 minutes, and 10 minutes during one set of three repetitions, with attention to variables such as set mean velocity (sMV), set mean force (sMF), and set mean power output (sMP). Even though the used device has confirmed reliability, there is a potential risk that analyzing single repetitions at higher velocities is less reliable [24]. Therefore, we analyzed the mean values based on three repetitions in sets [25].

### Participants

For two-way (2 groups × 5 measures) repeated measures analysis of variance (RM-ANOVA) considering within-between interaction required a sample size of at least 22 individuals based on a calculation using a power of 0.80, an effect size of 0.25 and an alpha of 0.05. Inclusion criteria included age between 18–25 years, absence of upper body musculoskeletal injury in the prior year, no medical contraindications to physical effort, continuous resistance training for at least three years, and no use of doping substances. The initial pool comprised 42 male subjects, with the final study sample consisting of 26 individuals (EG = 14; CG = 12), of whom 12 were excluded for not meeting inclusion criteria. Before the experimental session, one individual from the EG and three from the CG withdrew from the study for personal reasons unrelated to the investigation. Participants were instructed to avoid strenuous physical activity 72 hours before the experiment and maintain regular eating, drinking, and sleeping habits. The consumption of ergogenic substances like caffeine was prohibited 24 hours before measurements.

### Body morphology measurements

Body height was measured using an anthropometer (GPM Anthropological Instruments, DKSH Ltd., Zürich, Switzerland), and an InBody230 device (InBody Co., Ltd., Cerritos, CA, USA) recorded body mass. Body mass index (BMI) was obtained as body mass [kg]/body height [m^2^].

### Bench Press One-Repetition Maximum Assessment

The load-velocity relationship method was used to establish the BP 1RM [26]. The Vitruve VBT linear position transducer (Vitruve, SPEED4LIFTS S.L., Madrid, Spain) was used for measurements. The device reliability had been previously established [24]. The BP 1RM protocols employed a Smith machine to ensure consistency with the BPT. Participants underwent a general warm-up comprising a fiveminute treadmill run at 6 km/h, dynamic joint mobilization, three sets of 10–15 bodyweight push-ups, and five slam ball chest throws with 60-second rest intervals between sets. In the specific warm-up phase, participants executed 12–15 repetitions of an empty bar BP, emphasizing maximal concentric speed in each repetition with a hand grip set at 150% of the biacromial distance. Throughout these warmup phases, spotters were consistently present, overseeing and ensuring the proper execution of each set to guarantee repeatability. Subsequent sets included 10–12 repetitions at 40%1RM (mean velocity 1.0–1.2 m/s), followed by two to three sets of five repetitions at 60–80%1RM (mean velocity 0.5–0.75 m/s). Following the warmup, a progressive incremental test commenced with a load set at 80% 1RM for three reps (0.5 m/s mean velocity), with subsequent sets incorporating 5–10% load (kg) increases. As mean velocity dropped below 0.5 m/s, sets comprised two repetitions, and if it fell below 0.25 m/s, participants executed a single repetition. The test continued for a maximum of five sets or until failure, with a three to five-minute rest interval allowed between sets. Participants were instructed to control the lowering of the barbell. The tempo for a single repetition was established as 2/0/X/0 and controlled by the Vitruve VBT linear position transducer (Vitruve, SPEED4LIFTS S.L., Madrid, Spain). The eccentric phase was to last 2 seconds, with a first transition of 0 seconds. During the concentric phase, participants were encouraged to lift as quickly as possible (X) to overcome the load. A second transition was also 0 seconds. Bouncing was not permitted, and participants had to keep their lower back on the bench and their feet stable on the floor. Individuals received constant feedback on their performance [27].

### Conditioning Activity

The CA protocol for the EG included four three-second sets of hold isometric muscle action in BP with 70% 1RM and a rest interval of 60 seconds between sets. During the CA, the position of participants on the bench was the same as during the 1RM test. Participants had to keep their lower back on the bench and their feet stable on the floor. They lowered the barbell by bending their arms and actively held it in contact with their chest for 3 seconds. Next, with the assistance of spotters, the barbell was placed back on the hooks.

### Bench Press Throw Measurements

The BPT was performed at 30% 1RM on the Smith Machine, with the same body position as during the BP testing and CA as described above. The load was set according to previous studies [28]. To achieve maximal barbell velocity, participants were instructed to lower the barbell quickly without slowing down the movement to their chest and without bouncing the bar [29]. They were then to immediately press it upwards, releasing the bar at the peak of the motion of the upper extremities to throw it as high as possible with maximal engagement. To ensure safety, spotters caught the barbell before it reached the participant’s hands and returned it to the participant for the next attempt. Three repetitions per set were performed.

Measurements were taken at baseline (three minutes before CA) and 1, 4, 7, and 10 minutes after the PAPE protocol using a Vitruve VBT linear position transducer (Vitruve, SPEED4LIFTS S.L., Madrid, Spain). The directly registered parameters were set mean velocity (sMV) [m/s] and set mean power output (sMP) [W], whereas set mean force (sMF) [N] was calculated as sMF = sMP/sMV. A supervisor was consistently on hand to intercept and prevent the bar from descending too far, mitigating potential harm to the participants. Although the device employed has been verified for its reliability, there remains a possibility that the analysis of individual repetitions at higher velocities may not be reliable enough [24]. So, the analysis included mean values derived from three consecutive repetitions [25].

### Statistics

Data normality was confirmed using the Shapiro-Wilk test. Descriptive statistics were presented as means ± standard deviations (SDs) and 95% confidence intervals. The coefficient of variation was calculated for the set measures. A comparison using Student t-tests for independent samples was used to compare the EG and CG for baseline characteristics. Homogeneity of variance and data sphericity were assessed using Levene’s and Mauchly’s tests, respectively. Two-way repeated measures ANOVA tests were employed, and effect sizes (ES) were calculated as a partial eta-squared (η_p_^2^), interpreted as small (η_p_^2^ ≥ 0.01), medium (η_p_^2^≥ 0.06), or large (η_p_^2^≥ 0.14). A Bonferroni post hoc test was utilized, and Cohen’s d ES was calculated for significant differences and interpreted as small (< 0.20), medium (< 0.50), or large (> 0.80). The mediation analysis assesses both the total and specific indirect effects, indicating the influence of the predictor (CA) on the outcomes (sMP–dependent variable) through the proposed mediators (sMV and sMF). Baron and Kenny [15] introduced a four-step process: (1)examine statistically significant associations between the predictor and dependent variables, (2) assess the statistically significant association between the predictor and mediator, (3) demonstrate the mediator’s impact on the dependent variable, and (4) illustrate that the association between the predictor and dependent variables weakens when adjusting for the mediator in the model. If only one of the steps involving the mediator is statistically insignificant, there is no mediation. This approach is applicable in the examination of physical performance [16]. The significance level was set at a p-value of < 0.05 using Statistica13.0 software. The mediation analysis utilized the Jamovi’s Advanced Mediation Models 1.0.4 module (Jamovi, v.1.6, 2020).

## RESULTS

Table 1 shows the statistical characteristics of participants concerning group adherence. No statistically significant differences between participants from both groups were revealed (p > 0.05).

**Table 1:** Statistical description of study groups. Independent Student t-test results.

Variable	Experimental Group	Control Group	t	p

	Mean ± SD	Mean ± SD		
Age [years]	22.43 ± 2.93	21.75 ± 1.54	0.72	0.48
Resistance training experience [years]	5.71 ± 2.05	5.33 ± 1.30	0.55	0.59
Training volume [minutes/week]	310 ± 113.34	326.67 ± 102.72	-0.39	0.70
Body height [cm]	179.21 ± 8.13	178.58 ± 6.27	0.22	0.83
Body mass [kg]	87.29 ± 6.75	85.31 ± 8.85	0.65	0.52
Body Mass Index [kg/m^2^]	27.27 ± 2.48	26.75 ± 2.37	0.54	0.59
Relative strength [BP-1RM/Body mass]	1.40 ± 0.23	1.41 ± 0.19	-0.07	0.94

Table 2 presents the values of the analyzed parameters from consecutive minutes, with a maximal coefficient of variation (CV) for consecutive measurements provided. Results below 10% indicated a low variance in the measurements.

**TABLE 2:** Results achieved during the experiment in measured parameters.

Variable	Group	Baseline Mean ± SD maxCV [%]	1st Mean ± SD maxCV [%]	4th Mean ± SD maxCV [%]	7th Mean ± SD maxCV [%]	10th Mean ± SD maxCV [%]	Δ (Max-baseline)
Set Mean Velocity [m/s]	EG	1.20 ± 0.09	1.23 ± 0.09	1.26 ± 0.10*^EG−B^	1.26 ± 0.08*^EG−B^	1.27 ± 0.08*EG−B;1st	0.08 ± 0.04
		9.9%	7.8%	4.6%	6.2%	5.5%	

	CG	1.21 ± 0.06	1.22 ± 0.06	1.22 ± 0.07	1.22 ± 0.06	1.21 ± 0.07	0.03 ± 0.03
		9.2%	6.7%	9.1%	4.8%	3.0%	

Set Mean Force [N]	EG	272.6 ± 39.5	271.5 ± 38.3	272.6 ± 37.6	272.2 ± 38.1	271.5 ± 37.8	2.9 ± 4.2
		10.5%	7.1%	4.6%	6.2%	5.4%	

	CG	263.9 ± 21.4	262.4 ± 22.3	264.4 ± 22.6	264.1 ± 21.9	264.9 ± 20.8	3.5 ± 2.3
		9.2%	4.1%	7.6%	4.7%	3.0%	

Set Mean Power [W]	EG	327.9 ± 51.7	333.3 ± 46.9	342.6 ± 55.3*^EG−B;^	344.2 ± 54.4*^EG−B;^	345.2 ± 55.6*^EG−B;^	21.2 ± 11.9
		1.9%	7.5%	^1st^3.7%	^1st^5.2%	^1st^1.5%	

	CG	319.5 ± 22.1	318.5 ± 22.6	321.8 ± 22.9	321.7 ± 23.2	321.1 ± 23.4	6.6 ± 4.4
		6.8%	6.7%	5.8%	1.8%	1.6%	

* Statistically significantly higher results than indicated group EG-experimental group; CG – control group, in following minutes (B – baseline, 1^st^; 4^th^; 7^th^; 10^th^)

Two-way (group × time) repeated measures ANOVA revealed significant effects of group-time interaction for sMV (F = 6.10, η_p_^2^ = 0.20, p < 0.01). Therefore, the Bonferroni test was performed to make a detailed comparison. An increase in sMV was revealed at the 4^th^, 7^th^, and 10^th^ minute when compared to baseline (p < 0.01, ES = 0.36, ES = 0.48, ES = 0.45, respectively). Moreover, sMV in the 10^th^ minute after the CA was higher than in the 1^st^ minute (p = 0.0089, ES = 0.20). Analysis for sMF did not reveal any significant effect on the group, time, or interaction (F = 0.47, ηp2 = 0.01, p = 0.55). The intervention significantly affected sMP due to significant interactions of group times (F = 5.03, ηp2 = 0.17, p < 0.01). The Bonferroni post hoc test revealed significant improvements in sMP from the 4^th^, 7^th^, and 10^th^ minutes compared to baseline (p < 0.01) in all comparisons, with ES = 0.20, ES = 0.22, ES = 0.23, respectively. Also, the results from consecutive minutes were higher than the 1st-minute post-CA (p = 0.0459, ES = 0.14, p = 0.0068, ES = 0.16, p = 0.0019, ES = 0.20),

The last step was mediation analysis, in which **Δ** (max-baseline differences) were considered. Hypothesis 1 confirmed that the predictor – group adherence (presence of CA) – had an impact on the dependent variable – sMP – what was expressed as a total effect (β = 0.63, p < 0.001). The subsequent steps involved testing hypotheses 2 (a1 & a2) and 3 (b1 & b2), which revealed statistically significant associations for sMV. The predictor affected the sMV (mediator, path a1:β = 0.53, p = 0.002), and the mediator, in turn, was associated with the dependent variable, sMP (path b1:β = 0.69, p < 0.001). However, there was no significant effect observed for CA and sMF (path a2:β = -0.09, p = 0.613), so despite significant interaction between sMF and sMP (path b2:β = 0.35, p < 0.001), no mediation effect was revealed. The final step involved testing hypothesis 4, which indicated that the positive indirect effect of the CA on sMP (Indirect1: β = 0.37, p < 0.003) was significant when considering the mediation of sMV in that relationship, and no significant effect of sMF (Indirect2: β = -0.03, p = 0.613). The positive direct effect of the group would weaken when the sMV as a mediator was included in the model but was still significant, indicating a partial mediation (Direct:β = 0. 29, p = 0.001).

## DISCUSSION

The main aim of this study was to explore the mediating effect of force and velocity changes on power output enhancement during the BPT after isometric submaximal CA in trained males. The introduced CA protocol effectively enhanced upper body power output through increased barbell velocity while showing no improvement in force production capabilities. The positive effect occurred 4 minutes after CA and persisted in consecutive minutes.

Our analysis considers the effect on power output and deepens the issue through joint analysis of force and velocity changes, adding new insight into the PAPE effect. Therefore, our results are hard to discuss. No study has provided such an analysis; therefore, some generalizations can be made. A study by Esformes et al. [13], using isometric muscle action in a pushing manner with a 7-second contraction, showed improvements in peak power output in the BPT after CA without any significant changes in peak force production. This is similar to our observation; thus, the provided study partially relates to our research due to different protocols and approaches for kinematics expression. Research mainly focuses on reporting only power output and velocity, presenting varied results under different CA protocols. Hence, it is unclear how CA influences force [30, 31]. This was confirmed by Seitz and Haff [7], who observed that various types of CA may affect power output differently. Moreover, the above reports show that changes in power output after CA are not always related to velocity changes, and the assessment of force and velocity together is justified and could provide a more comprehensive picture of the PAPE effect.

For a deeper understanding and explanation of the observed results, it is necessary to consider the factors influencing the improvement of muscle contraction velocity. Contraction velocity relies on the activity of high-threshold motor units, their firing rate, and the frequency of stimulation [32]. Tillin and Bishop [8] postulate that the PAPE effect results in a greater velocity for a specific force (or vice versa). Therefore, it can be assumed that in our study, the enhancement in power output is attributed to an increased ability to generate higher velocity at the same level of force production. Engaging in isometric activities intensifies stimulation during CAs, and the sustained elevated nervous system activity post-concentric action leads to improvements in velocity and, consequently, power output during subsequent measurements after the CA. Not many studies have explored the relationship between isometric preconditioning and velocity, but some reports showed positive effects for velocity in a specific sports manner [14]. Methodological differences, however, hinder the ability to draw more profound connections.

In this term, another aspect for consideration is the relationship between force production and muscle velocity contraction. Higher contraction velocities reduce the ability to form cross-bridges between actin and myosin filaments within muscle fibers. This reduction in cross-bridge formation limits the amount of force that can be produced [33]. This phenomenon is part of the force-velocity relationship in muscle physiology, where muscles generate less force at higher contraction velocity [34]. Our observation suggests that the ability to form cross-bridges was maintained despite increased velocity with stable force production. This may be linked with increased myosin light chain phosphorylation, which leads to simplified crossbridge formation and maintained force production at higher velocity [35]. Thus, this effect on performance enhancement remains questionable [2].

Indeed, in our study, we addressed isometry at the transition point from eccentric to concentric action [29]. In this context, more effective energy use during the transition from eccentric to concentric phase muscle contraction was possible due to nervous system activation in this specific position that isometry can address; therefore, higher velocity was observed without higher demands for force production [36]. The effect of isometry is joint angle-specific; therefore, the adapted position of the barbell placed between eccentric and concentric movement could also contribute to the obtained effect [37].

Another consideration can be made for the viscoelastic properties of muscle-tendon units. Optimal stiffness can be related to better energy utility during eccentric-concentric action, which isometry can also affect [38]. Observations indicate that muscle tendon stiffness can increase after isometric training [39]. Thus, the acute effect of this after CA is unclear and requires further investigation [40]. Speculation on this topic is hindered by the lack of studies on the viscoelastic properties of the upper body after isometric CA.

The present study has limitations. The study design could be performed in a crossover manner, with the subjects being used as their own control. Additionally, the measurements did not consider individual force-velocity profiles, and assessment of viscoelastic properties would add more insight into the used isometric CA. Despite these limitations, the study offers strengths, particularly in providing unique insights into the acute effects of isometry on upper body performance capacities through deepened analysis. Based on our observation that specific improvements in velocity enhance power output, we question whether other types of CA might yield different results in terms of force production and velocity contraction and then in power output. To explore new possibilities in the PAPE effect, targeted activation approaches focusing on either force or velocity could be considered. First, the specific effects of different types of CA need to be identified. Therefore, future studies should explore other types of CA related to various muscle contractions and loads to verify this approach.

## CONCLUSIONS

The proposed PAPE protocol has shown efficacy in improving mean power output during the bench press throw through velocity enhancement with no significant impact on force production. It can be inferred that the observed enhancement in power output following the protocol is attributed to optimizing movement velocity with stable force production. Despite the static nature of isometry, it can provide positive results in velocity improvement [19]. Employing this recommended protocol as a direct preparatory measure prior to engaging in explosive training endeavors has the potential to acutely enhance upper body power output through the optimization of movement velocity. The positive effect can occur after 4 minutes of rest and persist in consecutive minutes. However, it is imperative to tailor the 70% of 1RM loads during the protocol, involving four three-second sets with one-minute rest intervals. Practitioners need to remember load individualization and rest periods, as these factors may vary among individuals.
